# Effect of surfactants and polymer composition on the characteristics of polyhydroxyalkanoate nanoparticles

**DOI:** 10.5599/admet.2723

**Published:** 2025-06-04

**Authors:** Aleksei Dorokhin, Sergei Lipaikin, Galina Ryltseva, Alexander Shabanov, Kristina Sapozhnikova, Tatiana Volova, Sergei Kachin, Ekaterina Shishatskaya

**Affiliations:** 1 Siberian Federal University, 79 Svobodny pr., Krasnoyarsk 660041, Russia; 2 L.V. Kirensky Institute of Physics, Siberian Branch of the Russian Academy of Sciences, 50/12 Akademgorodok, Krasnoyarsk 660036, Russia; 3 Institute of Biophysics SB RAS, Federal Research Center “Krasnoyarsk Science Center SB RAS”, 50/50 Akademgorodok, Krasnoyarsk 660036, Russia

**Keywords:** P3HB, P3HBV, polyvinyl alcohol, Tween 80, sodium deoxycholate, sodium dodecyl sulphate

## Abstract

**Background and purpose:**

Polyhydroxyalkanoates (PHAs) are biodegradable polyesters of bacterial origin that are actively studied as matrices for the preparation of nanoparticulate drug delivery systems. The most significant parameters affecting PHAs nanoparticles (NPs) characteristics are polymer composition and the type of surfactant used to stabilize the emulsion during NPs preparation. However, there are only a few studies in the literature investigating the effect of these factors on the characteristics of PHA NPs.

**Experimental approach:**

Blank poly(3-hydroxybutyrate) (P3HB) and poly(3-hydroxybutyrate-co-3-hydroxyvalerate) (P3HBV) NPs were produced and characterized in terms of their size, morphology and zeta potential. Poly(vinyl alcohol) (PVA) with various molecular weights (31-50 and 85-124 kDa), as well as Tween 20 (TW20), Tween 80 (TW80), sodium deoxycholate (SDC) and sodium dodecyl sulphate (SDS) were used as surfactants. For NPs that formed stable aqueous suspensions and had the most desirable characteristics (P3HB/PVA_31-50_ and P3HBV/PVA_31-50_), hemolytic activity and cytotoxicity to HeLa and C2C12 cells in vitro were determined.

**Key results:**

NPs of both P3HB and P3HBV obtained using PVA with the *M*_w_ of 31-50 kDa as a surfactant had regular spherical shape, uniform size distribution, average diameter of about 900 nm and zeta potential of -28.5 and -28.7 mV, respectively. PVA_85-124_, TW20 and TW80, as well as SDC and SDS as surfactants, did not show satisfactory results due to suspension gelation, formation of hollow NPs with irregular shape and poor resuspension after washing and freeze-drying, respectively. P3HB/PVA_31-50_ and P3HBV/PVA_31-50_ NPs did not have hemolytic activity and did not show pronounced cytotoxicity to HeLa and C2C12 cells in the concentration range from 10 to 500 μg mL^-1^, so these samples were regarded as safe and biocompatible.

**Conclusion:**

In this study, the effect of various non-ionic and anionic surfactants on the characteristics of P3HB and P3HBV NPs was investigated. PVA_31-50_ was found to be effective in producing NPs of both studied polymers with good biocompatibility and favorable characteristics, making them suitable for drug delivery applications. In contrast, other studied surfactants, *i.e.*, PVA_85-124_, TW20, TW80, SDC and SDS, require further investigation. The obtained findings may promote the development of novel PHA-based nanomedicines.

## Introduction

In recent years, there has been increasing interest in utilising biodegradable polymeric nanoparticles (NPs) to combat a plethora of diseases, such as cancer and bacterial infections. This nanoplatform represents a powerful tool in the field of medicine as it’s been found to successfully deliver antineoplastic drugs to target cells [1-4], suppress tumour growth in tumour-bearing animals *in vivo* [5-7] and overcome cancer drug resistance [8]. Thus, investigation of novel biodegradable polymeric nanovehicles has become an important facet of modern drug development.

Polyhydroxyalkanoates (PHAs) are a family of hydrophobic biodegradable polyesters of bacterial origin. These polymers gain much attention as a matrix for the preparation of various nanodrug formulations as they demonstrate excellent biocompatibility, biodegradability and controllable thermal and mechanical properties [9]. PHAs matrices were successfully used to encapsulate cytostatic [10-15] and antibacterial [16-18] drugs, anti-inflammatory agents of steroid and non-steroid nature [19], photosensitizers [20], antisense oligonucleotides [21], inclusion complexes [22], insulin [23,24], plant-derived essential oils [25], *etc*. However, despite intensive research in this area, the amount of experimental data on the influence of synthesis parameters and polymer composition on the characteristics of PHAs NPs is extremely limited.

At present, the emulsification solvent evaporation technique is one of the most widely employed methods for the preparation of PHAs NPs. In this method, surfactant plays an important role as it stabilizes the emulsion by decreasing the surface tension and primarily affects the NPs characteristics. A variety of compounds have been proposed for this purpose, including ionic and non-ionic. Among them, poly(vinyl alcohol) (PVA) is one of the most common non-ionic surfactants, since it’s nontoxic, biodegradable [27] and has a capacity to form relatively small particles with uniform size distribution [28]. For instance, PVA was found suitable to be utilized to obtain PHAs [29-31], poly(caprolactone) [32-34], and poly(lactic-co-glycolic acid) (PLGA) and its block copolymer with poly(ethylene glycol) (PEG) [35-37] particles of different sizes.

Another group of non-ionic surfactants are polysorbates, commercially available under the trade name Tween. Chemically, polysorbates are polyoxyethylated sorbitan esters that differ in the fatty acid residue. Polysorbates are widely used in the food, cosmetic, and pharmaceutical industries as they possess low toxicity and biodegradability. Furthermore, they are applied to stabilize polymeric NPs, since they show high surface activity, prevent protein adsorption and enhance NPs' ability to traverse biological barriers [28].

The cytotoxicity of surfactants varies depending on the chemical structure, but it generally decreases in the order: cationic surfactants > anionic surfactants ≥ zwitterionic surfactants > non-ionic surfactants [38]. Thus, among the many surfactants available, anionic compounds such as sodium dodecyl sulfate (SDS) and sodium deoxycholate (SDC) have found only limited application for NPs preparation due to safety concerns. It has been reported that SDS may cause irritation and a decrease in cell proliferation [39], so rigorous removal of the excessive surfactant from the resulting NPs is highly important. This effect may be a significant limitation for some systemic routes of administration, such as intravenous injection, where exposure to sensitive tissues and cells is unavoidable. However, the Food and Drug Administration (FDA) regards SDS as safe as an additive in the food industry [40], so oral administration of properly purified SDS-coated NPs may be considered acceptable [41]. According to literature, SDS was used to obtain PLGA particles of different sizes loaded with α-tocopherol [42], amoxicillin [43] and paclitaxel [44]. SDC also has a noticeable dose-dependent cytotoxic potential, but nanoparticulate drug delivery systems leveraging its ability to increase lipid membrane permeability and disrupt tight junctions in the epithelial lining may be suitable for mucosal and oral administration of bioactive compounds [45,46]. In addition, some studies revealed that anionic surfactants alone or in mixtures with other surfactants can be used to obtain NPs that possess the mean diameter much smaller than that of the NPs obtained using PVA [42,44].

Cationic surfactants have also not found wide application for the preparation of polymeric NPs due to their intrinsic membrane-disruptive activity. This effect is caused by the electrostatic interaction between the cationic nanoparticle surface and the negatively charged lipid membrane of cells. For instance, Hwang *et al.* [47] reported that common cationic surfactants cetyltrimethylammonium bromide (CTAB) and soyaethyl morpholinium ethosulfate (SME) in both nanoparticulate and free forms decrease cell viability of human neutrophils, induce membrane damage and the release of inflammatory mediators. Inácio *et al.* [48] also studied the cytotoxicity of cationic surfactants to mammalian cells and revealed that quaternary ammonium compounds induce not only membrane disruption but also mitochondrial dysfunction in MDCK II epithelial cells. The addition of non-ionic compounds such as PEG and PVA during NPs preparation may reduce cationic NPs’ cytotoxicity [49], but this approach has not been sufficiently studied.

Although several authors investigated the effect of preparation parameters on the size and stability of PHAs particles [50-52], to the best of our knowledge, a comprehensive study on the influence of various surfactants on the characteristics of PHAs NPs has not been reported.

The aim of this research is to investigate the influence of various non-ionic and anionic surfactants and polymer composition on the characteristics of PHAs nanoparticles.

## Experimental

### Materials

Microbial poly(3-hydroxybutyrate) (P3HB, 363 kDa) and poly(3-hydroxybutyrate-co-3-hydroxyvalerate) (P3HBV, 10.2 % of 3HV, 475 kDa) were produced at the laboratory of Biotechnology of New Biomaterials of Siberian Federal University in Krasnoyarsk, RF [53,54]. PVA (31-50 kDa, degree of hydrolysis 98-99 % and 85-124 kDa, degree of hydrolysis 99 %), dimethyl sulfoxide (DMSO) and standard antibiotic-antimycotic supplement were purchased from Sigma-Aldrich (USA). Tween 20 (TW20) was purchased from Panreac Applichem (Germany). Tween 80 (TW80) was purchased from Amresco (USA). SDC was purchased from Acros Organics (USA). SDS was purchased from Helicon (Russia). Dulbecco’s modified Eagle’s medium (DMEM) was purchased from Thermo Fisher Scientific (USA). Fetal bovine serum was purchased from HyClone (USA).

All reagents and solvents were of analytical grade and used as received without further purification. The water used was purified by Arium® Pro Ultrapure water system (Sartorius AG, Germany).

### Preparation of nanoparticles

P3HB and P3HBV nanoparticles were prepared using the single emulsion solvent evaporation technique [18,55]. In brief, 0.2 g of P3HB or P3HBV was dissolved in 20 mL of CHCl_3_ at 50 °C. The resulting solution was added dropwise to 100 mL of surfactant solution (0.5 %) in deionized water and mechanically stirred at 24000 rpm for 5 min (Heidolph SilentCrusher M, Germany). Then, the resulting emulsion was magnetically stirred at 1000 rpm for 24 h until the complete CHCl_3_ evaporation. The obtained nanoparticles were collected by centrifugation, washed 5 times with deionized water and freeze-dried.

### Yield of nanoparticles

Yield of the obtained nanoparticles (*Y*) was determined according to Equation (1) [56,57]:



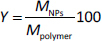

(1)


where *M*_NPs_ is a mass of the obtained NPs and *M*_polymer_ is a mass of the polymer used.

### Hydrodynamic particle size and zeta potential

The average hydrodynamic size and polydispersity index (PDI) of the obtained nanoparticles were determined by dynamic light scattering (DLS), while zeta potential was measured by electrophoretic light scattering (ELS) (Zetasizer Nano ZS, Malvern, UK) according to standard procedures described in [58-61]. Aqueous suspensions of each sample containing 2 mg of nanoparticles in 2 mL of deionized water were sonicated at 30 W for 1 min before the measurements (Misonix Sonicator S3000, USA). ZetaSizer Nano ZS software was used to analyze the results.

### Scanning electron microscopy

In order to assess the shape and surface morphology of the obtained nanoparticles, scanning electron microscopy (SEM) was used (Hitachi SU3500 and Hitachi TM4000, Japan). The samples were positioned on a specimen stub and sputter-coated with platinum (Leica EM ACE200, Germany) to increase conductivity and promote heat dissipation from the polymeric matrix.

### Determination of residual PVA

The amount of residual PVA associated with P3HB and P3HBV NPs obtained using PVA_31-50_ and PVA_85-124_ was determined spectrophotometrically by the method based on the formation of a blue-coloured complex between PVA and triiodide in the presence of boric acid described in [62,63]. In brief, 2 mg of freeze-dried NPs samples were treated with 2 mL of 0.5 M NaOH for 15 min at 60 °C. Then, 0.9 mL of 1 M HCl was added to each sample, and the volume was adjusted to 5 mL with deionized water. To each sample, 3 mL of a 0.65 M solution of B(OH)_3_, 0.5 mL of a solution of I_2_/KI (0.05 M/0.15 M), and 1.5 mL of deionized water were added. After incubation for 15 min, the optical density of the samples was measured at 690 nm. Calibration curves of PVA_31-50_ and PVA_85-124_ were prepared under the same conditions.

The amount of residual PVA, wt.% was calculated according to Equation (2) [63]:



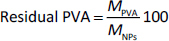

(2)


where *M*_PVA_ is a mass of PVA associated with NPs and *M*_NPs_ is a mass of the obtained NPs.

### Hemolytic activity

The hemolytic activity of P3HB/PVA_31-50_ and P3HBV/PVA_31-50_ was evaluated since NPs in these samples were aggregately stable in aqueous solutions. The determination of hemolytic activity was carried out in accordance with the procedure described in [64] with some modifications. In brief, the whole human blood containing EDTA as an anticoagulant was centrifuged at 3000 rpm for 10 min. Plasma and buffy coat were discarded, while red blood cells (RBCs) precipitate was collected, washed with normal saline and resuspended in normal saline to a concentration of 10^8^ cells mL^-1^. Then, 20 μL of P3HB/PVA_31-50_ or P3HBV/PVA_31-50_ colloidal solutions with various concentrations (to obtain the concentration of 10, 50, 100 and 500 μg mL^-1^ in wells) were added to 130 μL of the obtained RBCs suspension in a 96-well plate and incubated at 37 °C for 2 h. After incubation, RBCs were separated by centrifugation and the absorbance of haemoglobin in the supernatant at 415 nm was determined using an iMark Microplate Reader (Bio-Rad Laboratories, USA). RBCs in the positive control were treated with deionized water, which caused the complete osmotic lysis of the RBCs' membranes, while RBCs in the negative control were incubated with normal saline.

Hemolytic activity of P3HB/PVA_31-50_ and P3HBV/PVA_31-50_ was calculated according to the formula [64]:



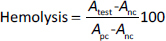

(3)


where *A*_test_ is the absorbance of samples incubated with NPs, *A*_pc_ is the absorbance of the positive control sample (RBCs incubated with deionized water) and *A*_nc_ is the absorbance of the negative control sample (RBCs incubated with normal saline).

### Cell culture

HeLa cervical cancer cells and C2C12 myoblast-like cells were cultured in Dulbecco’s modified Eagle’s medium (DMEM, Thermo Fisher Scientific, USA) supplemented with 10 % fetal bovine serum and 1 % penicillin-streptomycin. The cells were maintained at 37 °C in a 5 % CO_2_ humidified atmosphere and subcultured using 0.25 % trypsin-EDTA after reaching 70-80 % confluence.

### Cytotoxicity assessment

HeLa and C2C12 cells were planted at a density of 2×10^4^ cells per 1 cm^2^ in 96-well plates. After seeding and 24 h of incubation, the medium was replaced by fresh culture medium containing suspensions of P3HB/PVA_31-50_ or P3HBV/PVA_31-50_ with various concentrations (to obtain the concentrations of 10, 50, 100 and 500 μg mL^-1^ in wells) and the cells were incubated with NPs for 72 h. Then, the cell viability was determined using 3-(4,5-dimethylthiazol-2-yl)-2,5-diphenyltetrazolium bromide (MTT) assay as described elsewhere [65,66]. The absorbance of the resulting formazan dissolved in DMSO was recorded using an iMark Microplate Reader (Bio-Rad Laboratories, USA) after 4 h of incubation with 200 μL of MTT solution at 37 °C (*λ* = 550 nm). Cell viability was calculated relative to untreated cells according to the formula [67]:



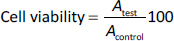

(4)


where *A*_test_ is the absorbance of samples incubated with NPs and *A*_control_ is the absorbance of the control sample.

To visualize live and dead cells, a LIVE/DEAD assay was performed using a ReadyProbes™ double staining kit (Thermo Fisher, USA) in accordance with the manufacturer’s protocol. Images were obtained using DM6000 B TL (BF) + Fluo «Leica» digital microscope (Leica Microsystems GmbH, Germany). Live and dead cells had blue and green fluorescence, respectively.

To estimate the cell monolayer surface area covered with NPs, an Eclipse Ti-U inverted microscope was used (Nicon, Japan). Cell images were analysed using Fiji open-source image processing software [68].

## Results and discussion

### NPs characteristics

To determine the average diameter and zeta potential of the obtained NPs, DLS and ELS methods were used, respectively. However, NPs of both polymers used, obtained using PVA_85-124_, TW20 and TW80, as well as SDC and SDS, were not suitable for DLS and ELS measurements due to solution gelation, NPs aggregation and poor resuspension after washing and freeze-drying, respectively. The characteristics of the NPs obtained are presented in Table 1.

**Table 1.** Characteristics of P3HB and P3HBV NPs obtained using various surfactants.

Sample	*Y* / %	*d* / nm	*P*dI	Zeta potential, mV
P3HB/PVA_31-50_	94.1	910.6	0.264	-28.5
P3HB/PVA_85-124_	102.3	Solution gelation, NPs aggregation, high residual surfactant content		
P3HB/TW20	87.5	Formation of porous NPs with an irregular shape and hollow polymeric “shells” tending to aggregate and precipitate		
P3HB/TW80	92.6			
P3HB/SDC	59.8	Poor resuspension after washing and freeze-drying		
P3HB/SDS	42.9			
P3HBV/PVA_31-50_	98.2	919.0	0.219	-28.7
P3HBV/PVA_85-124_	101.7	Solution gelation, NPs aggregation, high residual surfactant content		
P3HBV/TW20	81.0	Formation of porous NPs with an irregular shape and hollow polymeric “shells” tending to aggregate and precipitate		
P3HBV/TW80	85.9			
P3HBV/SDC	79.4	Poor resuspension after washing and freeze-drying		
P3HBV/SDS	53.5			

**Figure 1.**
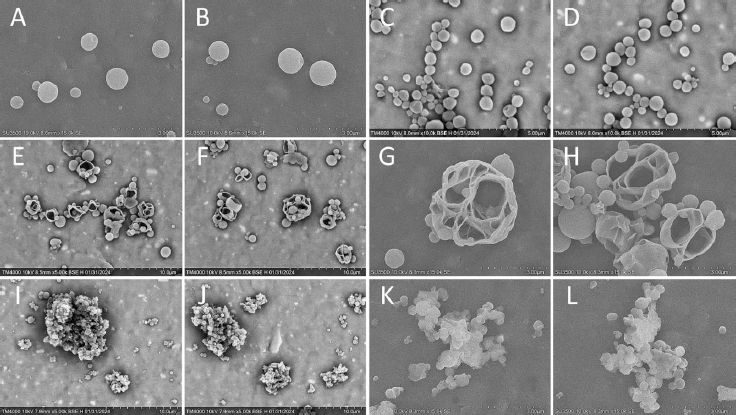
SEM images of the obtained NPs. (A) P3HB/PVA_31-50_. (B) P3HBV/PVA_31-50_. (C) P3HB/PVA_85-124_. (D) P3HBV/PVA_85-124_. (E) P3HB/TW20. (F) P3HBV/TW20. (G) P3HB/TW80. (H) P3HBV/TW80. (I) P3HB/SDC. (J) P3HBV/SDC. (K) P3HB/SDS. (L) P3HBV/SDS

P3HB and P3HBV NPs obtained using PVA_31-50_ and PVA_85-124_ had a smooth surface, regular spherical shape and the mean diameter of approximately 900 nm (Figure 1). However, P3HB/PVA_85-124_ and P3HBV/PVA_85-124_ NPs were prone to gel formation due to high residual surfactant content even after multiple washings with deionized water. At high NPs concentrations, complete gelation of the solution was observed, while diluted NPs suspension underwent local gelatinization, leading to NPs aggregation. According to the literature, PVA molecules cannot be removed entirely during the washing procedures due to the formation of an interconnected network with the polymer [63] and therefore remain associated with the surface of polymeric nanoparticles [69]. During NPs formation, the residual hydrophobic vinyl acetate groups of a partially hydrolyzed PVA serve as an anchoring site on the hydrophobic polymer surface, while more hydrophilic fully hydrolyzed segments are exposed to the aqueous phase [70]. As PVA_85-124_ used in this research has a high degree of hydrolysis, the formation of the gelled network between NPs via strong hydrogen bonds between hydroxyl groups of PVA molecules occurred [71], making it impossible to measure NPs size and zeta potential via DLS and ELS methods, respectively.

Residual PVA influences the physical and chemical properties of NPs' surface, such as zeta potential and hydrophobicity and can modulate cellular uptake. The amount of PVA associated with P3HB/PVA_31-50_, P3HBV/PVA_31-50_, P3HB/PVA_85-124_ and P3HBV/PVA_85-124_ NPs increased with the increase of its molecular weight (Table 2). These results are in accordance with those obtained by Azizi *et al.* [72]. They reported that PVA with a high molecular weight (146-186 kDa) covered bovine serum albumin-loaded PLGA NPs more than PVA with the *M*_w_ of 13-23 and 70-87 kDa.

**Table 2.** Residual PVA associated with P3HB and P3HBV NPs.

Sample	Residual PVA, % (w/w)
P3HB/PVA_31-50_	1.5±0.5
P3HB/PVA_85-124_	7.0±0.6
P3HBV/PVA_31-50_	1.7±0.4
P3HBV/PVA_85-124_	7.5±0.9

P3HB/PVA_31-50_ and P3HBV/PVA_31-50_ NPs had zeta potential values of -28.5 and -28.7 mV, respectively, so colloidal solutions of these samples were regarded as stable [61]. Moreover, residual PVA on the NPs surface is beneficial as it provides the additional steric stabilization of the NPs [73].

Samples prepared using both Tween 20 and Tween 80 contained porous NPs with an irregular shape and hollow polymeric “shells” tending to aggregate and precipitate (Figure 1). Similar surface morphology was shown by different authors for curcumin-loaded P3HB NPs prepared using 0.1 % aqueous solution of Tween 80 [74], as well as blank P3HB NPs obtained using 0.1 % aqueous solution of Span 20 [52]. Formation of such particles may be attributed to water occlusion during CHCl_3_ evaporation or double emulsion formation. Tween 80 has a hydrophilic-lipophilic balance (HLB) of 15.0 and promotes the formation of O/W emulsions [75]. However, it has been shown that TW80 can facilitate the formation of W/O emulsions in the mixture with Span 80 (HLB 4.3), even though the HLB value of this mixture is not as low as that for pure Span 80 [76].

NPs obtained using both SDC and SDS were prone to poor resuspension after washing and freeze-drying, even after the addition of another surfactant (Tween 80 or PVA) solution in the NPs suspension and extended sonication. According to SEM, P3HB/SDC, P3HBV/SDC, P3HB/SDS and P3HBV/SDS NPs had spherical shape and the average diameter was less than 500 nm (Figure 1). Similar reduction in particle size compared to particles prepared using PVA as a surfactant was observed by Esim *et al.* [77].

These particles formed large aggregates, and only a few individual particles were detected. Such a tendency to agglomerate was shown for PLGA microspheres obtained using anionic surfactant dioctyl sodium sulfosuccinate [43], some samples of zein NPs obtained using SDC [78], as well as PLGA-PEG NPs obtained using sodium cholate that differs from SDC by only the 7α-hydroxyl group [79]. We assume that this may be due to the fact that low-molecular anionic surfactants, unlike PVA, do not provide a sufficient steric stabilization of nanoparticles. Thus, during the washing procedures, the SDC and SDS molecules are removed from the NPs surface, which leads to a decrease in the electrostatic repulsion and aggregation and precipitation of the NPs. In addition, the yield of nanoparticles obtained using anionic surfactants was unsatisfactory for both polymers used, which may also be due to strong aggregation of particles during the washing stage. To increase the NPs’ yield, a mixture of anionic and non-ionic surfactants may be used. This approach exploits not only the steric stabilization of NPs by PVA moieties but also the capacity of anionic surfactants to produce NPs of small sizes [44].

In this work, no tangible influence of the 3-hydroxyvalerate content on the characteristics of the nanoparticles was revealed.

### Hemolytic activity

**Figure 2.**
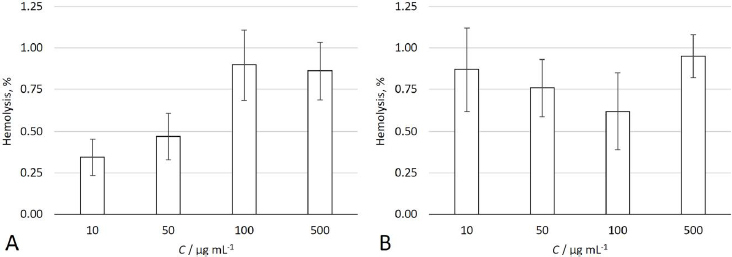
Hemolytic activity of P3HB/PVA_31-50_ (A) and P3HBV/PVA_31-50_ (B) at different NPs concentrations

To estimate the hemocompatibility of the NPs that form stable colloidal solutions and possess the most desirable morphology (P3HB/PVA_31-50_ and P3HBV/PVA_31-50_), the hemolytic activity of these samples at various concentrations (10, 50, 100 and 500 μg mL^-1^) was determined. According to literature, NPs with less than 5% hemolytic activity are considered safe and hemocompatible [80,81]. As shown in Figure 2, the percentage of hemolysis induced by both P3HB/PVA_31-50_ and P3HBV/PVA_31-50_ was less than 2 % at all concentrations used. So, according to this criterion, NPs in the samples P3HB/PVA_31-50_ and P3HBV/PVA_31-50_ do not show membrane-disrupting activity. These results are in accordance with those obtained by Chen *et al.* [82]. They reported that P3HB-PEG-P3HB NPs have negligible hemolytic activity even at a concentration of 120 μg mL^-1^.

Due to solution gelation, NPs aggregation and poor resuspension, NPs obtained using PVA_85-124_, TW20, TW80, SDC and SDS were unsuitable for the *in vitro* experiments, since NPs in these samples did not form stable colloidal solutions.

### In vitro cytotoxicity

**Figure 3.**
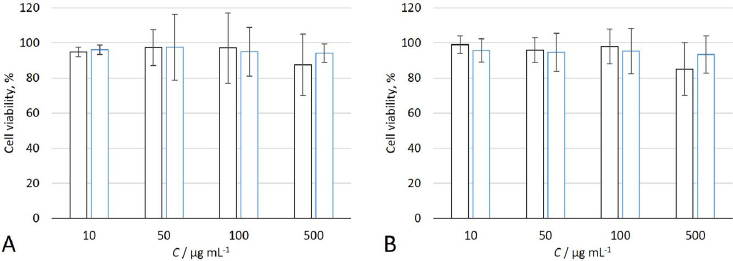
Viability of HeLa (black) and C2C12 (blue) cells treated with P3HB/PVA_31-50_ (A) and P3HBV/PVA_31-50_ (B) at different NPs concentrations

**Figure 4.**
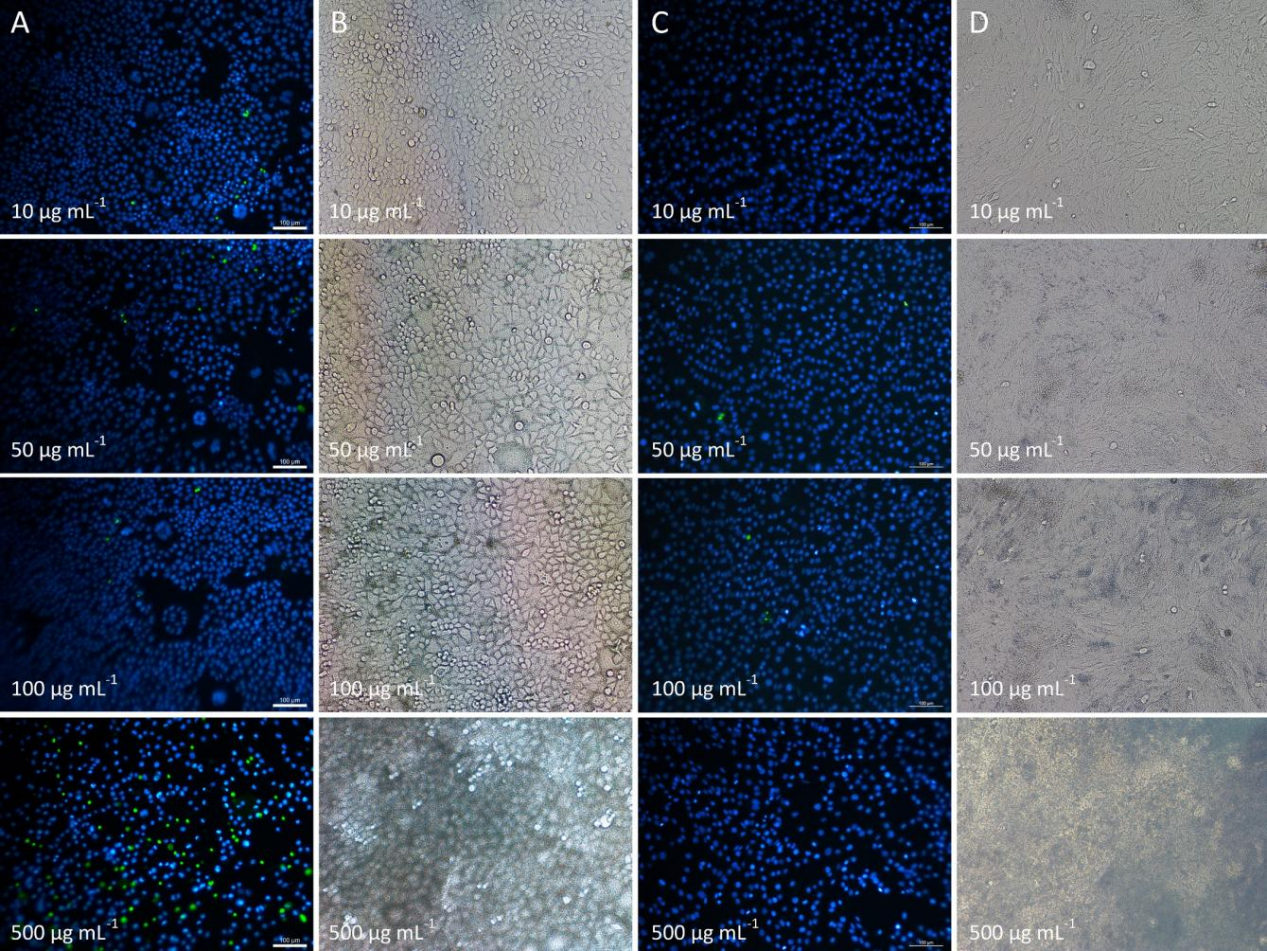
Images of HeLa (A, B) and C2C12 (C, D) cells treated with P3HB/PVA_31-50_ NPs at different concentrations obtained using fluorescent and optical microscope. Live and dead cells had blue and green fluorescence, respectively. At the concentrations of 10, 50, 100 and 500 μg mL^-1^ P3HB/PVA_31-50_ NPs covered 8, 21, 55 and more than 90 % of cell monolayer, respectively. Data for P3HBV/PVA_31-50_ are not presented

In this study, cytotoxicity of P3HB/PVA_31-50_ and P3HBV/PVA_31-50_ NPs to HeLa cervical cancer cells and C2C12 myoblast-like cells was evaluated. As shown in Figure 3, at the concentrations of 10, 50 and 100 μg mL^-1^, NPs of both studied samples did not influence HeLa cell viability. However, at the relatively high concentration of 500 μg mL^-1^, a slight increase in the amount of dead cells was observed, as NPs covered more than 90 % of the cell monolayer, impairing transport of nutrients to cells and hindering cell proliferation (Figure 4). At the same time, C2C12 cells were less sensitive to NPs exposure, and no significant decrease in cell viability was observed at any NPs concentration. NPs predominantly accumulated in the area of cell junctions, which may be caused by the complex of adhesion, bending and protrusion of at least two cell membranes interacting with NPs and trapping them in this area [83].

Biodegradable polymeric nanoparticles, in general, are considered safe and biocompatible and show cytotoxic effects only at relatively high concentrations. Hu *et al.* [84] reported that poly(3-hydroxybutyrate-co-3-hydroxyvalerate-co-3-hydroxyhexanoate) NPs did not affect the cell viability of Jurkat (human T lymphocytes) and Raji (human B lymphoblastoid) cells even at the concentration of 5 mg mL^-1^. Similar results were obtained by Masood *et al.* [12]. P3HBV NPs showed only ≤22.49 % A549 (pulmonary epithelium) cancer cell inhibition at 250 μg mL^-1^ concentration after 72 h of incubation. Silva *et al.* [85] reported that PLGA NPs are slightly cytotoxic to Y-79 human retinoblastoma cells only at the NPs concentration of 32 μmol L^-1^. Cell viability was decreased to 85-90 % of the control, so the studied NPs were regarded as safe for drug delivery applications. Xiong *et al.* [86] also investigated PLGA NPs cytotoxicity and reported that these nanoparticles showed no cytotoxic effects to RAW264.7 (murine macrophage) and BEAS-2B (human bronchial epithelial) cells at the NPs concentrations ranging from 10 to 300 μg mL^-1^, while TiO_2_ NPs at the concentration of 300 μg mL^-1^ significantly decreased BEAS-2B cell viability.

## Conclusions

In this study, the effect of various non-ionic and anionic surfactants on the characteristics of P3HB and P3HBV NPs was investigated. NPs obtained using PVA with *M*_w_ of 31-50 kDa revealed a smooth surface, regular spherical shape, a size of about 900 nm, appropriate zeta potential, negligible hemolytic activity and low cytotoxicity to HeLa and C2C12 cells. Thus, PHAs NPs obtained using PVA_31-50_ are a promising carrier for the preparation of various nanodrug formulations. However, NPs prepared using PVA_85-124_ tended to undergo hydrogel formation due to high residual surfactant content even after multiple washings with deionized water. TW20 and TW80 also showed unsatisfactory results as surfactants owing to the formation of hollow polymeric “shells” tending to aggregate and precipitate in aqueous solutions. Nevertheless, such hollow particles, while unsuitable for creating drug delivery systems, may serve as a promising candidate for tissue engineering purposes. NPs obtained using anionic surfactants SDC and SDS were significantly smaller, but further studies are required to ensure their aggregation stability.
